# Factors associated with body mass index in children and adolescents: An international cross-sectional study

**DOI:** 10.1371/journal.pone.0196221

**Published:** 2018-05-02

**Authors:** Edwin A. Mitchell, Alistair W. Stewart, Irene Braithwaite, Rinki Murphy, Robert J. Hancox, Clare Wall, Richard Beasley, the ISAAC Phase Three Study Group

**Affiliations:** 1 Department of Paediatrics: Child and Youth Health, Faculty of Medicine and Health Sciences, The University of Auckland, Auckland, New Zealand; 2 School of Population Health, Faculty of Medicine and Health Sciences, The University of Auckland, Auckland, New Zealand; 3 Medical Research Institute of New Zealand, Wellington, New Zealand; 4 Department of Medicine, Faculty of Medicine and Health Sciences, The University of Auckland, Auckland, New Zealand; 5 Department of Preventive & Social Medicine, Dunedin School of Medicine, University of Otago, Dunedin, New Zealand; 6 Discipline of Nutrition and Dietetics, Faculty of Medicine and Health Sciences, The University of Auckland, Auckland, New Zealand; McMaster University, CANADA

## Abstract

**Background:**

The increasing prevalence of overweight and obesity in childhood has implications for their future health. There are many potential contributors to overweight and obesity in childhood. The aim was to investigate the association between postulated risk factors and body mass index (BMI) in children and adolescents.

**Methods:**

Secondary analysis of data from a multi-centre, multi-country, cross-sectional study (ISAAC Phase Three). Parents/guardians of children aged 6–7 years completed a questionnaire about their child’s current height and weight, and the postulated risk factors. Adolescents aged 13–14 years reported their own height and weight and answered questions about the postulated risk factors. A general linear mixed model was used to determine the association between BMI and the postulated risk factors. Imputation was used if there were missing responses for 3 or fewer explanatory variables.

**Results:**

65,721 children (27 centres, 15 countries) and 189,282 adolescents (70 centres, 35 countries) were included in the final analyses. Many statistically significant associations were identified, although for most variables the effect sizes were small. In children birth weight (for each kg increase in birth weight the BMI increased by +0.43 kg/m^2^, p<0.001), television viewing (5+ hours/day +0.33 kg/m^2^ vs. <1 hour/day, p<0.001), fast food (≥3 times/week +0.16 kg/m^2^ vs. never, p<0.001) vigorous physical activity (3+ hours/week 0.071 kg/m^2^ vs. never, p = 0.023) and maternal smoking in the first year of life (+0.13 kg/m^2^, p<0.001) were associated with a higher BMI in the adjusted model. Nut consumption (≥3 times/week -0.11 kg/m^2^ vs. never, p = 0.002) was associated with a lower BMI. Early life exposures (antibiotics, paracetamol and breast feeding) were also associated with BMI. For adolescents statistically significant associations with BMI and were seen with maternal smoking (+0.25 kg/m^2^, p<0.001), television viewing (5+ hours/day +0.23 kg/m^2^ vs. <1 hour/day, p<0.001), fast food (≥3 times/week -0.19 kg/m^2^ vs. never, p<0.001), vigorous physical activity (3+ hours/week 0.047 kg/m^2^ vs. never, p<0.001) and nuts (≥3 times/week -0.22 kg/m^2^ vs. never, p<0.001).

**Conclusions:**

Although several early life exposures were associated with small differences in BMI, most effect sizes were small. Larger effect sizes were seen with current maternal smoking, television viewing (both with higher BMI) and frequent nut consumption (lower BMI) in both children and adolescents, suggesting that current behaviours are more important than early exposures. Although many variables may influence BMI in childhood, the putative factors studied are not of sufficient magnitude to support major public health interventions.

## Introduction

The increasing prevalence of overweight and obesity in childhood has major implications for their current and future health [1]. This is not an issue just for high-income countries, but is also recognised as a problem for low and middle income countries [2]. At the simplest level the cause of childhood overweight and obesity is an energy imbalance between calories consumed and calories expended. A number of factors are underlying this including a dietary shift towards increased intake of energy-dense foods that are high in fat and sugars, and decreased physical activity levels due to increasing sedentary behaviour, such as television viewing, changes in transportation, and increasing urbanization [3].

Although there have been many studies of risk factors for overweight and obesity, these have been mainly conducted in high-income countries. Furthermore, most studies have categorised body weight (underweight, normal weight, overweight and obese). However, there is evidence that the whole distribution of body mass index (BMI) is increasing, but with a disproportionate increase at higher BMI levels [4].

We have previously examined in detail the association between BMI and television viewing (5), birth weight [6], breastfeeding [7], fast food [8], antibiotics [(9], paracetamol [10], maternal and paternal smoking [11], vigorous physical activity [12] and food [13] in a large international survey of children and adolescents. The aim of this paper was to investigate the association between all these risk factors and BMI in children and adolescents.

## Methods

The methods have been described in detail previously [5]. This study is a secondary analysis of the data from the International Study of Asthma and Allergies (ISAAC) Phase Three study. ISAAC is an international multicentre cross-sectional study investigating the prevalence and risk factors for symptoms of asthma, rhinoconjunctivitis and eczema, and the role of risk factors [14]. Data for ISAAC Phase Three was collected between 2000 and 2003 and involved 6–7 year old children and 13–14 year old adolescents chosen from a random sample of schools in a defined geographic area. The questionnaire was in two parts, the core questionnaire on symptoms of asthma, rhinoconjunctivitis and eczema, and an additional questionnaire regarding putative risk factors for asthma. The risk factor questionnaire was optional and many centres chose to administer only the core questionnaire. Questionnaires were translated into the local language and back translated to ensure accuracy [15].

The children’s questionnaires were completed by their parents. Adolescents completed the questionnaires themselves. As adolescents were unlikely to know the answer of the early life exposures, a more limited number of questions were asked of this age group.

### Outcome measure

Height and weight were reported by parents of the children and were self-reported by the adolescents. In some centres height and weight were measured objectively (20% of children and 23% of adolescents), although there were no standardised instructions for this. BMI was calculated (weight (kg)/height (m)^2^).

### Explanatory variables

In both age groups the following questions were asked: paracetamol (acetaminophen) use in the last year (yes/no), television viewing (less than 1 hour, 1 hour but less than 3 hours, 3 hours but less than 5 hours and 5 hours or more per day), vigorous exercise (categorised as never or occasionally, once or twice per week, three or more times a week), current maternal smoking (yes/no), current paternal smoking (yes/no) and the following food items in the last year: fast food, vegetables, fruit, pulses and nuts (categorised as never or occasionally, once or twice per week, three or more times a week).

The following questions were asked only of the parents of the children: birth weight as a continuous measure (kg), antibiotics in first year of life (yes/no), paracetamol (acetaminophen) in the first year of life (yes/no), maternal smoking in the first year of life (yes/no) and breastfed (yes/no). The supplementary table (S1 Table) gives the wording of the questions.

### Data cleaning

To eliminate likely erroneous BMI data while preserving as much data as possible, we applied the following methods:

- For children in each centre, those in the top and bottom 0.5% of weights and heights (n = 1,391), and those with heights less than 1.0 metre were excluded (n = 346). Children with BMI less than 9 kg/m^2^ or greater than 40 kg/ m^2^ were excluded (n = 215).
- For adolescents in each centre, those in the top and bottom 0.5% of weights and heights (n = 3,712), and those with heights less than 1.25 m were excluded (n = 904). Adolescents with BMI less than 10 kg/ m^2^ or greater than 45 kg/ m^2^ were excluded (n = 360).

### Statistical analyses

BMI was assessed separately for each age group using a general linear mixed model with centre as a random effect and gross national income (GNI) for each country, the individual’s age, sex and measurement type (reported/measured) and each of the explanatory variable separately (referred to as univariable analyses). To this model all the explanatory variables were added (referred to as multivariable analysis). SAS (v9.3, SAS Institute, Cary, NC, USA) was used. Imputation was used if there were missing responses for 3 or fewer explanatory variables. Adjustment for multiple testing was not made as each of the variables examined were associated with BMI in the literature or within this dataset when examined individually [5–13].

## Results

**Fig 1:**
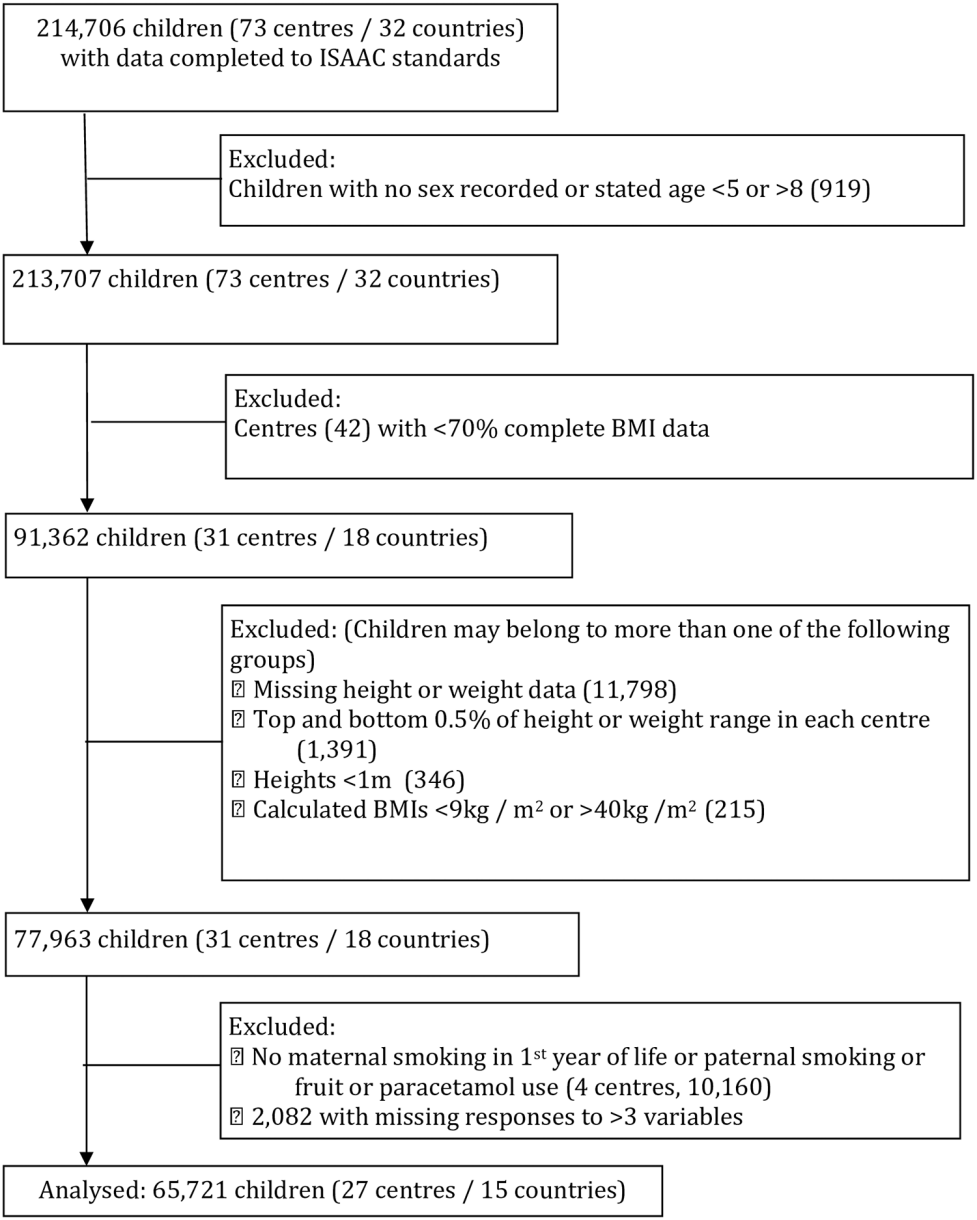
Flow of participants through the study. Children (6–7 years of age).

**Fig 2:**
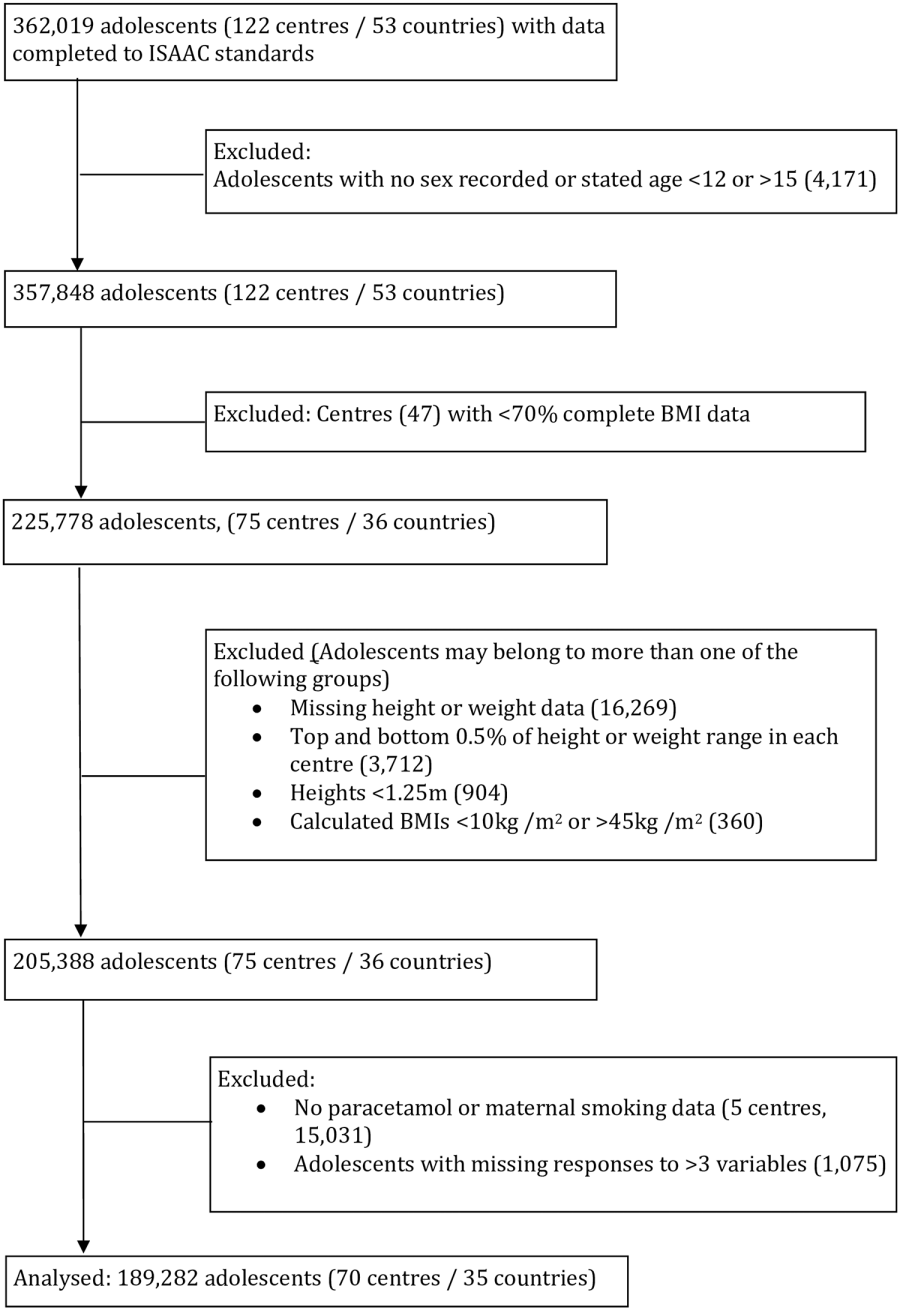
Flow of participants through the study. Adolescents (13–14 years of age).

Following sequential application of the exclusion and data cleaning criteria described above, 65721 children (27 centres/15 countries) and 189282 adolescents (70 centres/35 countries) were included in the final analysis (Fig 1—children and Fig 2—adolescents). In the adolescent age group two variables (current paternal smoking and pulses) were excluded from the analysis due to missing data (14.5% and 8.1% respectively).

The mean height and weight of children was 122.3 cm and 24.1 kg, and for adolescents 159.7 cm and 50.5 kg. The mean BMI was 16.1 kg/m^2^ and 19.8 kg/m^2^ respectively.

Table 1 shows the difference in BMI (kg/m^2^) between children and adolescents not exposed to the postulated risk factor and those exposed to that risk factor. The findings of the univariable and multivariable analyses are similar. Of the 15 explanatory variables in the model for children, for only one variable (nuts) did the p-value change from non-significance (p = 0.077) in the univariable model to significant in the multivariable model (p = 0.002). Of the 8 explanatory variables in the model for adolescents one variable (fruit) changed from significant (p<0.001) in the univariable model to marginal significance in the multivariable model (p = 0.052).

**Table 1:** Association between BMI (+/- kg/m^2^, (SE) and p = value) and explanatory variables for children (6–7 years) and adolescents (13–14 years).

	Children (6–7 years)		Adolescents (13–14 years)	
	Univariable*	Multivariable¶	Univariable*	Multivariable¶
**Birth weight (per kg)**	**p<0.001**	**p<0.001**		
**Continuous**	**0.448 (0.019)**	**0.433 (0.018)**		
**Breastfed**	**p = 0.021**	**p = 0.001**		
**No**	**ref**	**ref**		
**Yes**	**-0.062 (0.027)**	**-0.080 (0.025)**		
**Antibiotics in first year of life**	**p = 0.001**	**p = 0.027**		
**No**	**ref**	**ref**		
**Yes**	**0.071 (0.022)**	**0.047 (0.021)**		
**Paracetamol in first year of life**	**p<0.001**	**p = 0.027**		
**No**	**ref**	**ref**		
**Yes**	**0.080 (0.023)**	**0.050 (0.023)**		
**Paracetamol in the last year**	**p = 0.257**	**p = 0.007**	**p<0.001**	**p<0.001**
**None**	**ref**	**ref**	**ref**	**ref**
**> = 1/year**	**-0.024 (0.029)**	**-0.066 (0.027)**	**-0.062 (0.019)**	**-0.077 (0.018)**
**> = 1/month**	**-0.064 (0.039)**	**-0.110 (0.036)**	**0.072 (0.022)**	**0.058 (0.021)**
**Television viewing (hours/day)**	**p<0.001**	**p<0.001**	**p<0.001**	**p<0.001**
**<1**	**ref**	**ref**	**ref**	**ref**
**1–3**	**0.244 (0.028)**	**0.207 (0.025)**	**0.116 (0.024)**	**0.113 (0.023)**
**3–5**	**0.367 (0.036)**	**0.290 (0.033)**	**0.174 (0.026)**	**0.174 (0.025)**
**5+**	**0.374 (0.054)**	**0.328 (0.049)**	**0.212 (0.028)**	**0.227 (0.027)**
**Vigorous exercise (hours/week)**	**p = 0.050**	**p = 0.023**	**<0.001**	**p<0.001**
**Never**	**ref**	**ref**	**ref**	**ref**
**1–2**	**0.056 (0.027)**	**0.056 (0.025)**	**0.184 (0.020)**	**0.193 (0.019)**
**3+**	**0.070 (0.032)**	**0.071 (0.029)**	**0.022 (0.022)**	**0.047 (0.022)**
**Maternal smoking in the first year of life**	**p<0.001**	**p<0.001**		
**No**	**ref**	**ref**		
**Yes**	**0.183 (0.032)**	**0.128 (0.035)**		
**Current maternal smoking**	**p<0.001**	**p = 0.009**	**p<0.001**	**p<0.001**
**No**	**ref**	**ref**	**ref**	**ref**
**Yes**	**0.174 (0.028)**	**0.084 (0.032)**	**0.262 (0.019)**	**0.245 (0.019)**
**Current paternal smoking**	**p<0.001**	**p<0.001**		
**No**	**ref**	**ref**		
**Yes**	**0.134 (0.022)**	**0.090 (0.021)**		
**Fast food (times per week)**	**p<0.001**	**p<0.001**	**p<0.001**	**p<0.001**
**Never**	**ref**	**ref**	**ref**	**ref**
**1–2**	**0.150 (0.028)**	**0.126 (0.026)**	**-0.163 (0.017)**	**-0.158 (0.017)**
**3+**	**0.201 (0.054)**	**0.159 (0.048)**	**-0.202 (0.025)**	**-0.189 (0.024)**
**Vegetables (times/week)**	**p = 0.287**	**p = 0.928**	**p = 0.065**	**p = 0.537**
**Never**	**ref**	**ref**	**ref**	**ref**
**1–2**	**-0.021 (0.037)**	**-0.010 (0.035)**	**-0.007 (0.025)**	**0.007 (0.024)**
**3+**	**-0.049 (0.037)**	**-0.009 (0.036)**	**-0.042 (0.025)**	**-0.011 (0.024)**
**Fruit (times/week)**	**p = 0.839**	**p = 0.653**	**p<0.001**	**p = 0.052**
**Never**	**ref**	**ref**	**ref**	**ref**
**1–2**	**-0.018 (0.046)**	**-0.030 (0.042)**	**-0.078 (0.030)**	**-0.052 (0.029)**
**3+**	**-0.025 (0.044)**	**-0.012 (0.041)**	**-0.120 (0.029)**	**-0.067 (0.028)**
**Nuts (times per week)**	**p = 0.077**	**p = 0.002**	**p<0.001**	**p<0.001**
**Never**	**ref**	**ref**	**ref**	**ref**
**1–2**	**-0.037 (0.024)**	**-0.054 (0.023)**	**-0.172 (0.017)**	**-0.137 (0.017)**
**3+**	**-0.077 (0.039)**	**-0.113 (0.036)**	**-0.275 (0.026)**	**-0.224 (0.025)**
**Pulses (times/week)**	**p = 0.321**	**p = 0.114**		
**Never**	**ref**	**ref**		
**1–2**	**-0.001 (0.028)**	**0.007 (0.027)**		
**3+**	**0.039 (0.034)**	**0.055 (0.033)**		

* Adjusted for gross national income (GNI) for each country, the individual’s age, sex, measurement type (reported/measured)

^¶^ Adjusted for gross national income (GNI) for each country, the individual’s age, sex, measurement type (reported/measured) and all the explanatory variables

This analysis identified many statistically significant associations, although for most the effect sizes were small. In children the major risk factor for greater BMI was birth weight. For each additional kg in birth weight the BMI was 0.43 kg/m^2^ higher (p<0.001). The next major risk factor was television viewing (5+ hours/day vs. <1 hour/day, 0.33 kg/m^2^, p<0.001). Fast food (≥3 times/week + vs. never) and maternal smoking in the first year of life (yes vs. no) increased BMI by 0.16 kg/m^2^ and 0.13 kg/m^2^ respectively. Paracetamol in the last year (> = 1/month vs. never) and eating nuts (≥3 times/week vs. never) were associated with a lower BMI (-0.11 kg/m^2^ and -0.11 kg/m^2^). Other factors which were significantly associated with BMI but the effect size was <0.1 kg/m^2^ were breastfeeding (-0.08 kg/m^2^), antibiotics in the first year of life (0.05 kg/m^2^), paracetamol in the first year of life (0.05 kg/m^2^), vigorous exercise (0.07 kg/m^2^), current maternal smoking (0.08 kg/m^2^) and current paternal smoking (0.09 kg/m^2^). Eating vegetables, fruit and pulses (≥3 times/week vs. never) were not associated with BMI.

For adolescents statistically significant associations with BMI were seen with television viewing (5+ hours/day vs. <1 hour/day, 0.23 kg/m^2^, p<0.001), maternal smoking (+0.25 kg/m^2^, p<0.001), fast food (≥3 times/week vs. never, -0.19 kg/m^2^, p<0.001), nuts (≥3 times/week vs. never, -0.22 kg/m^2^, p<0.001), paracetamol in the last year (0.06 kg/m^2^) and vigorous exercise (0.05 kg/m^2^). Eating vegetables (≥3 times/week vs. never) was not associated with BMI. Eating fruit (≥3 times/week vs. never) was marginally associated with BMI (-0.07 kg/m^2^; p = 0.052).

**Fig 3:**
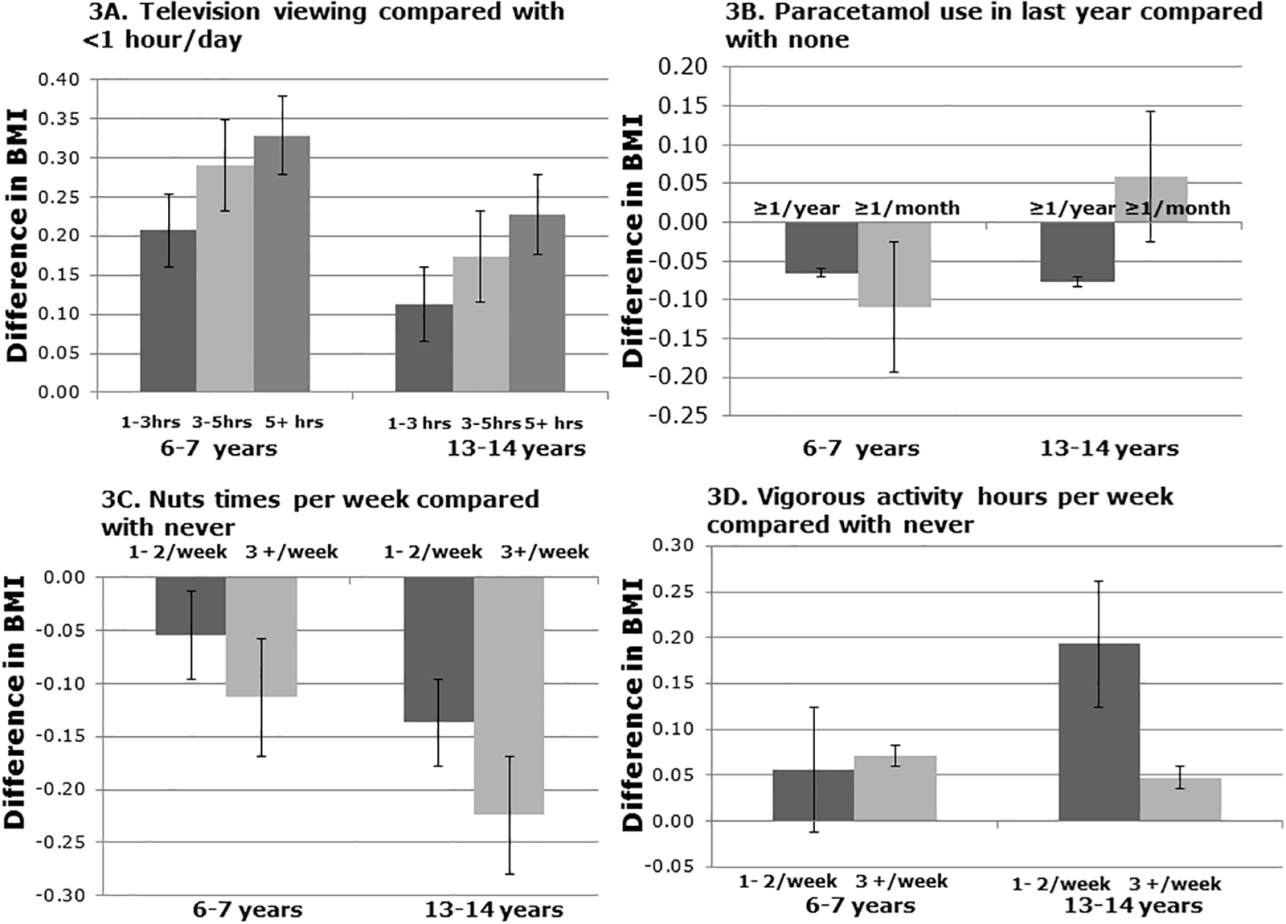
Graphical representation of change in BMI with television viewing (3A), paracetamol in the last year (3B), nuts (3C), and vigorous exercise (3D) in children (6–7 years of age) and adolescents (13–14 years of age).

**Fig 4:**
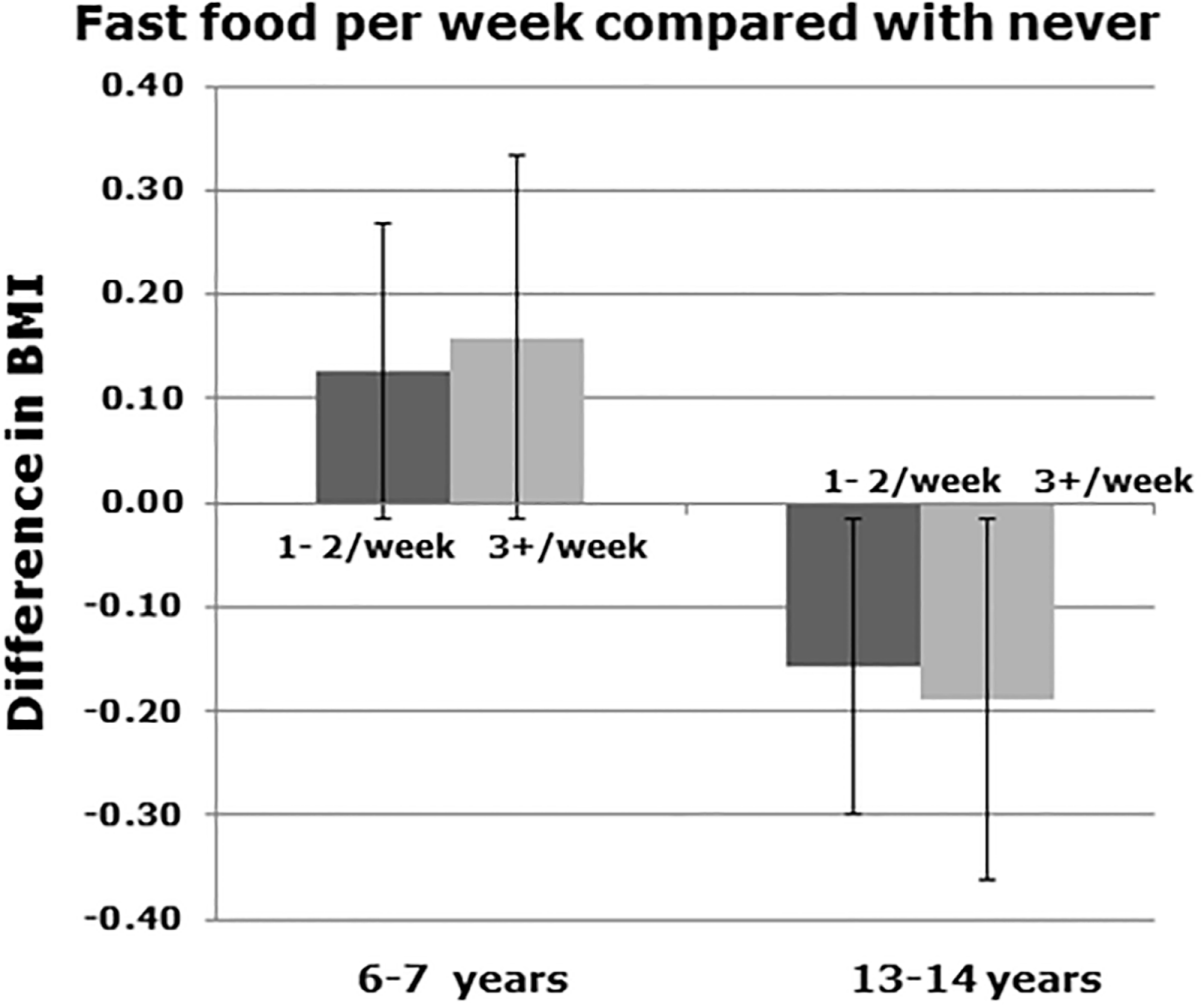
Graphical representation of change in BMI with fast food in children (6–7 years of age) and adolescents (13–14 years of age).

In both age groups television viewing (Fig 3A) and frequent consumption of nuts (Fig 3C) showed a dose effect. Paracetamol in the last year (Fig 3B) and vigorous exercise (Fig 3D) showed a dose effect in children but not in adolescents. Fast food (Fig 4) showed a dose effect but in a different direction for the two age groups.

## Discussion

In this secondary analysis of a very large dataset (65,721 children and 189,282 adolescents) we examined 15 and 8 putative risk factors for BMI in children and adolescents respectively. Despite finding many statistically significant associations with BMI, the effect sizes were small with many less than 0.1 kg/m^2^, which equates to a change of less than 0.15 kg body weight for children and 0.26 kg for adolescents who are at the mean height and weight in this study population.

In children the major risk factors for a higher BMI were birth weight, television viewing, maternal smoking in the first year of and eating fast food. Eating nuts was associated with a lower BMI. Because the questionnaire was self-completed, fewer questions were asked of adolescents as they were unlikely to know the answers relating to exposures in early life. In adolescents the major risk factors for a higher BMI were current maternal smoking and television viewing. Eating nuts was also associated with a lower BMI. Unexpectedly, eating fast food in this age group was associated with a lower BMI.

Caution needs to be applied when interpreting the results as the cross sectional design of this study does not allow determination of causality. In general there was a consistency in the findings between children and adolescents. In both age groups there was a positive association between television viewing and BMI and there was evidence that the more television was watched the higher the BMI. This provides support for a causal inference, but it remains possible that television viewing is simply a marker for other lifestyle factors, such as diet, lack of physical activity and socioeconomic factors, although we did adjust for vigorous physical activity and to a more limited extent diet. Our findings are consistent with other cross sectional [16] and longitudinal studies [17]. In addition the findings are consistent with a randomised controlled trial where an intervention designed to reduce children’s television viewing resulted in a significant reduction in BMI compared with the control group [18].

In both age groups parental smoking was associated with higher BMI values, which is consistent with the literature [19, 20]. Maternal smoking in the first year of life and current maternal smoking were both risk factors for higher BMI in children, but is not clear which age of exposure is more important because smoking at one time period is likely to be strongly associated with smoking at other time periods. Furthermore, it is unclear whether in utero tobacco smoke exposure, which was not measured in this study, has a direct effect [21] or whether the association is due to confounding due to differences in diet or other unhealthy lifestyles [22].

Surprisingly we found that vigorous physical activity in both age groups was positively associated with BMI, although the effect size was small and a dose effect was not consistent. Another study of 136,000 youths found that in 29 of 33 participating countries greater physical activity participation was associated with a lower risk of being overweight [23]. Why our study found the opposite association is not clear, however an increase in BMI due to muscle gain from exercise might explain provide an explanation. Alternatively this could be a result of reverse causation, where children and adolescents are exercising more due to their elevated BMI or due to a reporting bias with greater recall of exercise among those with a higher BMI.

In both age groups eating nuts were associated with a lower BMI, and there was a dose effect. Nuts contain high levels of fats and protein and thus are high energy density foods [24] and have sometimes been considered to be unhealthy but recent evidence suggests nut intake has been associated with the reduction of risk factors for chronic diseases [25]. However, our finding is not consistent with a meta-analysis of 33 clinical trials which found that diets enriched with nuts compared with control diets did not increase or decrease BMI or waist circumference [26].

There were two instances when the findings in children and adolescents were in the opposite direction. In children eating fast food was positively associated with BMI, but in adolescents was negatively associated with BMI. The positive association in children was expected and is consistent with other studies [27]. However the adolescent findings were unexpected. As adolescents reported their own eating behaviours they may have under reported fast food intake, particularly if they were overweight. Alternatively those that are overweight or obese may avoid fast food as a strategy for weight management.

Paracetamol use in the last year in children was associated with a reduced BMI but an inconsistent pattern in adolescents. Given that the association of paracetamol use with BMI differs by GNI [10], this is likely to reflect confounding by indication rather than any direct effect of paracetamol on BMI.

We have previously shown that there was a positive linear relationship between birth weight and BMI in children. There was no evidence of a J- or U-shaped association. Although the magnitude of the association of birth weight and BMI was the largest of any variable studied (0.43 mg/kg^2^ change for each kg of birth weight), this does not lead to a public health intervention because lower birth weights have other undesirable consequences. Nevertheless, it is important to understand the influence of in utero growth on the risk for childhood overweight/obesity.

There were a number of other statistically significant associations but the effect sizes were small. Among these was breastfeeding, which was associated with a small trend to lower BMIs. This small and clinically insignificant effect size contrasts with the emphasis that some authorities have given to breastfeeding as one of the key measures to prevent childhood obesity [28]. Our findings support other research that suggests that this emphasis is misplaced [29, 30].

The negative findings are also important. Many dietary guidelines from around the world recommend increased intakes of fruits and non-starchy vegetables as they are associated with a reduced risk of cardiovascular disease and cancer. However the evidence that they reduce obesity is not well established, especially in children and adolescents [31, 32]. Despite this, interventions to prevent or treat obesity often include the recommendation to increase fruit and vegetable intake in the hope that they will replace energy-dense foods. In our study the intake of vegetables, fruit and pulses were not associated with BMI.

It was noticeable that the multivariable results were similar to the univariable findings. This suggests that the risk factors examined were independent of each other.

Strengths of the study include the large number of participants (65,721 children and 189,282 adolescents) and that the participating centres were from countries with differing socioeconomic development, thus enabling the results to be generalised. The weakness of this study is that height and weight were reported by the parents/guardians for children and self-reported for the adolescents. In some centres height and weight were measured, but there was no standard protocol for doing this. Self-reported height and weight might introduce measurement error, and this would decrease the likelihood of finding and association. Ideally body composition would have been measured, but this was not feasible given the size of the study and inclusion of centres from low-middle income countries, many of whom had not undertaken such surveys previously. Lack of measurement precision is recognised and might influence the results towards the null. Furthermore, the exposure questions were not validated. As this was a secondary analysis of data that had already been collected this was not possible. However, it should be noted that the original ISAAC study was designed as a simple questionnaire that could be conducted in centres/countries that were resource poor and had limited research experience. Validation in such a setting would be impractical. A further limitation was that 7% of participants had missing height or weight. These data are may not be missing randomly, for example, overweight adolescents might decline to answer the weight question.

This study has shown that increasing birthweight and early life exposures, such as breastfeeding, antibiotic and paracetamol exposure were associated with higher BMI values. This provides some support for the programming hypothesis in fetal and early life where persisting changes in the body structure and function are caused by environmental stimuli [33]. Associations were also observed with current maternal smoking, television viewing and frequent nut consumption (reduced BMI) in both children and adolescents, suggesting that current behaviours are also important determinants of BMI. While each of these variables may influence BMI, they are not of sufficient magnitude to support major public health interventions. However, we recognise that establishing healthy behaviours in childhood may be important for later life.

## Supporting information

S1 Table Wording of the questions. (DOCX)
