# Supplementary material for: Factors associated with body mass index in children and adolescents: An international cross-sectional study
Source: PLoS One. 2018 May 2;13(5):e0196221. doi: 10.1371/journal.pone.0196221 (PMC5931641; doi:10.1371/journal.pone.0196221)
Supplement: S1 Table — (DOCX) [file pone.0196221.s001.docx]

**Wording of the questions.**

How much does your child / do you weigh?

How tall is your child / are you?

In the past 12 months, how often, on average did your child / did you eat fruit?

In the past 12 months, how often, on average did your child / did you eat vegetables (green and root)?

In the past 12 months, how often, on average did your child / did you eat pulses (peas, beans, lentils)?

In the past 12 months, how often, on average did your child / did you eat nuts?

In the past 12 months, how often, on average did your child / did you eat fast food/burgers?

How many times a week does your child / do you engage in vigorous physical activity long enough to

make him / her / you breathe hard?

During a normal week, how many hours a day (24 hours) does your child / do you watch television?

In the first 12 months of your child’s life, did you usually give paracetamol (e.g. [local trade name(s) for paracetamol]) for fever?

In the past 12 months, how often, on average, have you given your child / have you taken paracetamol (e.g. [local trade name(s) for paracetamol])?

In the first 12 months of life, did your child have any antibiotics?

What was the weight of your child when he / she was born?

Was your child breastfed?

Does your (child’s) mother (or female guardian) smoke cigarettes?

Does your (child’s) father (or male guardian) smoke cigarettes?

Did your child’s mother (or female guardian) smoke cigarettes during your child’s first year of life?
